# Innovative approaches to service integration addressing the unmet needs of irritable bowel syndrome patients and new approaches for the needs of IBS patients

**DOI:** 10.3389/fmed.2022.998838

**Published:** 2022-11-16

**Authors:** Maurizio Gentile, Vincenzo De Luca, Roberta Patalano, Daniela Laudisio, Giovanni Tramontano, Sonja Lindner-Rabl, Lorenzo Mercurio, Elena Salvatore, John Farrell, Regina Roller-Wirnsberger, Lutz Kubitschke, Maria Triassi, Annamaria Colao, Maddalena Illario, Vigour Consortium

**Affiliations:** ^1^Dipartimento di Medicina Clinica e Chirurgia, Università degli Studi di Naples Federico II, Naples, Italy; ^2^Dipartimento di Sanità Pubblica, Università degli Studi di Naples Federico II, Naples, Italy; ^3^Unità Operativa Semplice Ricerca e Sviluppo, Azienda Ospedaliera Universitaria Federico II, Naples, Italy; ^4^EIP on AHA Reference Site Collaborative Network, Brussels, Belgium; ^5^Department of Internal Medicine, Medical University of Graz, Graz, Austria; ^6^Dipartimento di Scienze Biomediche Avanzate, Università degli Studi di Naples Federico II, Naples, Italy; ^7^Empirica Gesellschaft für Kommunikations- und Technologieforschung GmbH, Bonn, Germany; ^8^United Nations Educational, Scientific and Cultural Organization (UNESCO) Chair for Health Education and Sustainable Development, Federico II University, Naples, Italy

**Keywords:** irritable bowel syndrome, mHealth, digital health, nutrition, physical activity

## Abstract

**Background:**

Irritable bowel syndrome (IBS) is a common multifactorial condition that affects the large intestine and is characterized by chronic and relapsing abdominal pain and altered bowel habit. IBS is due to a combination of genetic, environmental and dietary factors. It's usually a lifelong problem very frustrating to live with and can have a big impact on quality of life, as single-agent therapy ra.

**Objective:**

To analyze the approaches and solutions that address the social and health unmet needs of patients with IBS.

**Design:**

A quantitative-qualitative approach was adopted in the current study to identify and specify key digital solution and high impact user scenarios applied to IBS patients, through an adaptation of the “Blueprint on Digital Transformation in Health and Care in an Ageing Society” persona methodology.

**Settings:**

Digital health solutions bring the potential of supporting health interventions through mobile apps, wearable devices, telemedicine.

**Patients:**

A Survey was administered to a group of patients in an anonymous form, and no need for Medical Ethical Committee approval was identified.

**Interventions:**

The theoretical elaboration IBS personas was developed through an interdisciplinary Focus Group, which also mapped the pathway for the patient's management.

**Main outcome:**

Three main needs were identified to be met to improve IBS patient's lifestyle: access to psychological support, mHealth solutions supporting diet and adapted physical activity, and home-based digital health support. mHealth intervention has been identified for diet adherence, physical exercise and psychological well-being. The process has been mapped and adapted to integrate the new solutions into the care pathway.

**Limitation:**

Further research is needed to evaluate how mHealth services enable IBS patients to manage their conditions and improve their quality of life.

**Conclusion:**

The person-centered approach was implemented through a multidisciplinary Focus group that enabled the identification of the need for a mHealth intervention.

## Introduction

Irritable bowel syndrome (IBS) is a common multifactorial condition that affects the large intestine and is characterized by chronic and relapsing abdominal pain and altered bowel habit. The symptoms of IBS can overlap with those of other functional gastrointestinal disorders (FGIDs), indeed up to a third of patients show more than one feature, suggesting a common underlying etiology (1). IBS is due to a combination of genetic, environmental and dietary factors. Symptom-based diagnostic criteria (2) include symptom severity and frequency (sporadic, daily) and stool characteristics (3), that are also used to classify patients with IBS according to Rome IV criteria, depending on their predominant bowel habit: diarrhea-predominant (IBS-D), constipation-predominant (IBS-C), mixed diarrhea/constipation (IBS-M), and unclassified (IBS-U). The parameters for the diagnosis of IBS are based on abdominal pain and altered bowel habit in the absence of specific pathology (4). However, bloating, passage of mucus and incomplete rectal evacuation, nausea, back-ache, tiredness, which are common and troublesome symptoms in people with IBS, are not among the Rome criteria (5).

IBS is usually a lifelong problem very frustrating and can have a big impact on quality of life, as single-agent therapy rarely relieves bothersome symptoms for all patients. It is a prevalent disorder that greatly reduces patients' quality of life (QOL) and adversely affects the medical economy (6). A recent epidemiological survey using the Rome IV criteria revealed that the prevalence of IBS in the general population globally is 4.1% (7), with a higher prevalence of 7.7 in Italy (8), where this scenario was further worsened by the Covid-19 pandemic.

The impact of IBS on the individual, in terms of quality of life, and on health-care delivery and society, in terms of economic costs, are considerable (9).

Anxiety and depression are frequent mental health disorders worldwide (10), that have been increasing in the past 20 years and show a global prevalence of 12.9% (11). The emergence of the COVID-19 pandemic has created an environment where many determinants of poor mental health are exacerbated. Indeed, two COVID-19 impact indicators, specifically daily SARS-CoV-2 infection rates and reductions in human mobility, were associated with increased prevalence of major depressive disorder, and a total prevalence was 4802·4 cases (4108·2 to 5588·6) per 100 000 population. Altogether, a major depressive disorder caused 49·4 million (33·6 to 68·7) DALYs and anxiety disorders caused 44·5 million (30·2 to 62·5) DALYs globally in 2020 (12).

In IBS a visceral hypersensitivity related to altered processing of sensory stimuli along the brain-gut axis has been shown, especially in several brain areas like the insula. Psychiatric disorders were seen in 84.4% of IBS patients compared to 41.5% in controls. Major psychiatric disorders seen in our patients were general anxiety disorders (30%) and depression (28 %) (13). The interactions between biology, behavior, cognitive processes, and environment can directly influence gut functioning (motility, visceral pain levels), contributing to IBS symptoms (14). Meditation and yoga improve cognitive processes, reducing the intensity of anxiety, depression and, more generally, the ability to manage emotions (15, 16).

Like for anxiety and depressive disorders, abnormal brain network synchrony that correlates with self-bodily consciousness, measured by hypochondriasis and enteroception evaluation scales has been evidenced (14, 17). Meditation and yoga-like practice have been suggested as lifestyle practices that help to mitigate anxiety and IBS symptoms (15) and so they may be used to reduce drug prescriptions and improve quality of life. Digital health solutions bring the potential of supporting health interventions through mobile apps, wearable devices, telemedicine, or video games (16, 18). Mobile apps can promote a healthy lifestyle, encourage individuals to be healthier and more active, and offer smartphone-based personalized interventions for diet and physical activity coaching (19). The objective of this study was to propose some models of the policies, approaches, and solutions that address the social and health unmet needs of patients with irritable bowel syndrome.

## Methods

A mixed qualitative-quantitative method was adopted in the current study to identify and specify key digital solutions and high-impact user scenarios applied to IBS patients, through an adaptation of the “Blueprint on Digital Transformation in Health and Care in an Ageing Society” methodology (20) to the outpatient colo-proctology clinic of the Department of Clinical Medicine and Surgery, referral for IBS patients. The personas approach is a patient-centred methodology to design or identify key digital solutions and usage scenarios, which have a high impact on an individual's specific and unmet needs (21, 22). A persona is defined as a single, specific hypothetical/fictitious person who represents a segment of the population (23) with a realistic name, a face, and a description of their character (needs, goals, hopes, dreams, and attitudes). The Blueprint personas also include behavioral characteristics, which could affect both short-term and long-term success with interventions directed toward managing a disease or adopting wellness (24), for example, a persona's trust or lack of trust in care professionals, their self-management capabilities, and specific details about their character (e.g., being prone to aggressive behavior or having the tendency to reject outside support). In the present study, a collaborative approach has been implemented to outline a Blueprint persona affected by irritable bowel syndrome and identify the digital solutions to integrate in their care and cure. A Survey was administered to a group of patients in an anonymous form, and no need for Medical Ethical Committee approval was identified, so as no data were used, neither were references made to a specific single patient. The patients received an informed consent about the inclusion critheria and the use of data for privacy.

INCLUSION CRITHERIA were: (1) Age (18-70), (2) All gender, (3) No psychiatric illness under surveillance or treatment, (4) No previous proctological treatment, (5) No IBD, (6) No affiliation to other nutritional programme.

Theoretical elaboration of prototypes representative of IBS patients was developed through an interdisciplinary Focus Group (25, 26) involving:

- n.1 Proctology specialist;
- n.1 Clinical Psychologist specialist;
- n.2 Nutritionists;
- n.2 Experts in Digital Health;
- n.1 Expert in Healthcare organization.

**Figure 1:**
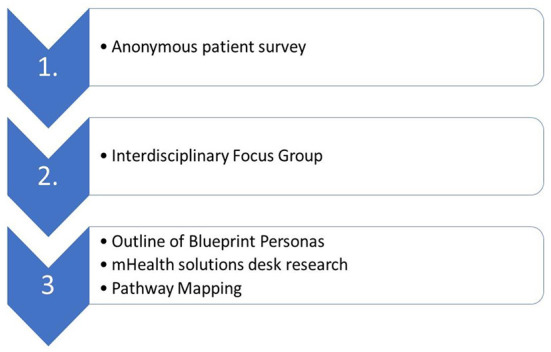
Care process.

Based on the IBS Personas, the Focus Group also mapped the pathway for the management of an IBS patient and implemented desk research on mHealth solutions addressing IBS personas unmet needs. The Focus Group divided the management of IBS into events (activities, interventions, or staff interactions), and analyzed mHealth solutions. These events (outpatient visits and lifestyle modification) were interpreted as a new patient care process (Figure 1).

## Results

### IBS patients

The Blueprint Survey was administered to a group of 50 patients diagnosed with IBS. The group included 22 males and 28 females, with a mean age of 41.33 (range 18–70). 34 of these (15M, 19F) were active workers, 6 were retired (5M, 1F), 5 unemployed and 5 were household. Diagnosed according to Rome IV criteria, 27 patients suffering IBS with predominant constipation (12M, 15F), 12 with predominant diarrhea (2M, 10F), 11 patients had mixed bowel habits (2M, 9F). A group of 12 patients showed a positive test for lactose intolerance and were excluded from the study. All patients were treated with a change of the diet increasing the amount of fibers and fluids intake. All patients were advised to exercise regularly. In 12 out 50 a FODMAP diet was administered and in 38 a high fiber rate diet was prescribed with medical therapy for constipation (macrogol, psyllium) or diarrhea (loperamide, anticholinergic). In three cases antidepressant (SSRI) were associated. 13 patients (10M, 3F) complained for more complex associated symptoms and they needed surgery. 8 complete or single node hemorrhoidectomy and 5 lateral internal sphincterotomy were performed (Figure 1).

### IBS personas

**Figure 2:**
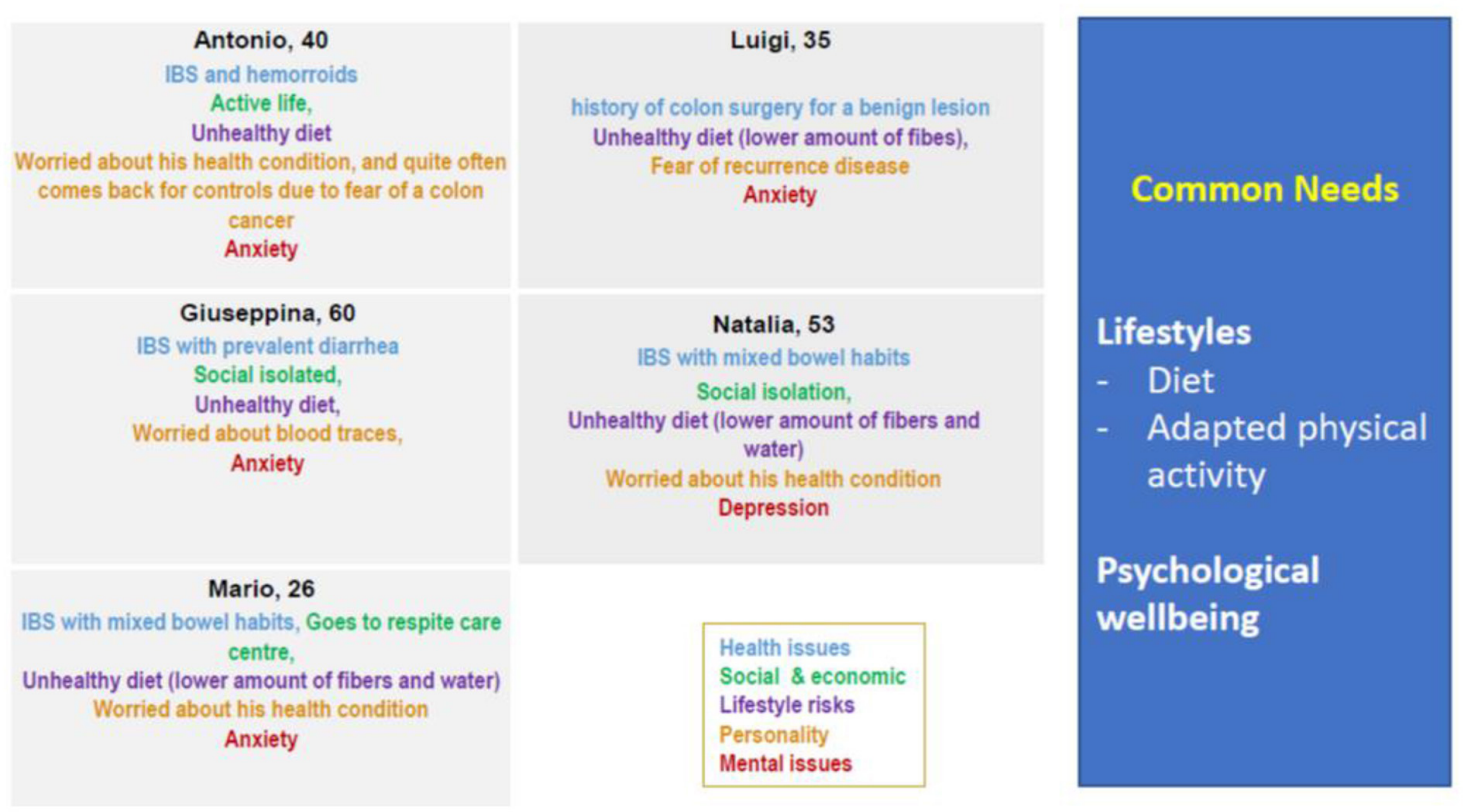
IBS Personas key features.

Based on the characteristics of the population of 50 patients included in the study, the Focus Group developed 5 Personas, following the Blueprint methodology (Figure 2).

**Figure 3:**
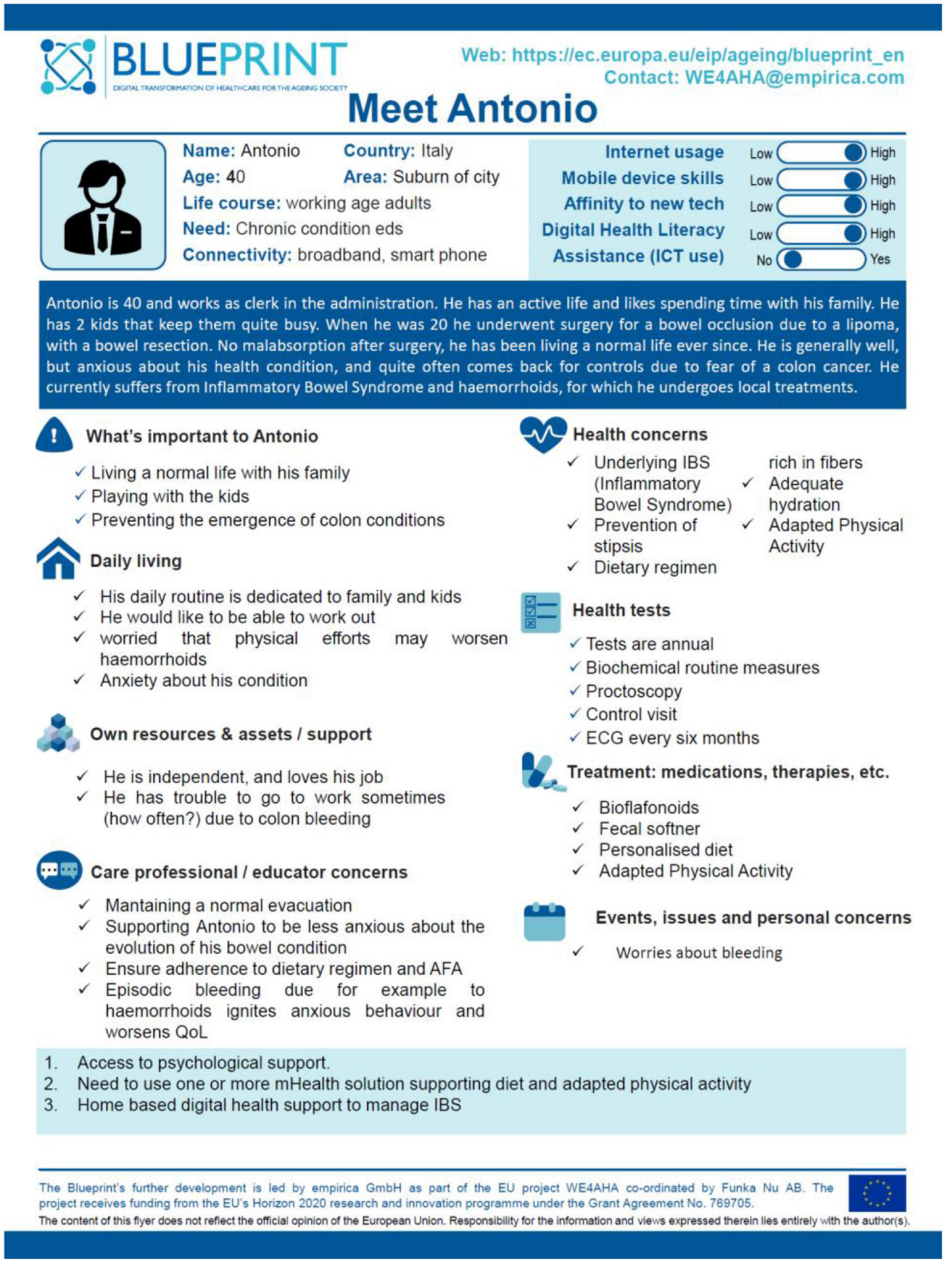
IBS Blueprint personas poster.

To identify Digital Health models that had an intervention strategy for IBS treatment and management, a persona, Antonio, has been developed who somewhat embodies all the recurring issues and needs in these patients. Age, general information, personal and life, hobbies, working condition, health concerns, and health needs have been taking in consideration to design a global picture. More in details, Antonio is a 40 years-old man with an active life. When adolescent he underwent surgery for a bowel occlusion due to a lipoma, with a bowel resection. He is generally well, but anxious about his health condition, and quite often comes back for controls due to fear of a colon cancer. He currently suffers from IBS and haemorrhoids, for which he undergoes local treatments. Three main needs were identified to be met to improve Antonio's lifestyle: access to psychological support, need to use one or more mHealth solutions supporting diet and adapted physical activity, and home-based digital health support to manage IBS (Figure 3).

### mHealth interventions

Mobile apps for the treatment of IBS may support psychoeducation on the etiology of IBS and the effectiveness of Cognitive Behavioral Therapy (CBT) in treatment. These mobile apps train the patients on various CBT strategies to mitigate the impact of IBS in daily life, including relaxation training, cognitive restructuring and catastrophizing, exposure exercises to reduce avoidance, and behavioral experiments (27).

Despite the differences in the symptoms, all IBS Personas might take advantage of a support for adherence to diet, physical exercise, and psychological wellbeing. These common, unmet needs informed the Focus Group search for mHealth solutions.

Lifestyles are the first level of intervention for IBS patients. Personalized coaching solutions may allow patients to prevent and avoid a sedentary lifestyle and receive useful and comprehensive long-term coaching. Personalized coaching mobile apps allow patients to receive advice on nutrition and other lifestyles (such as smoking, drinking, drug abuse and others), in line with personal preferences (28). Interactive coaching makes it possible to measure progress and classify a patient's behavior in order to identify possible warnings to be communicated to users.

Healthy nutrition coaching is based on the patient's meal intake data. The medical staff establishes the nutritional plan and the goals to be achieved. Other parameters such as body weight and weight variations, as well as concomitant diseases the patient may have, are considered in setting the goals. It is important to take the patient's preferences into account. The solution monitors the patient's adherence to nutritional prescriptions (29).

Similarly, physical activity coaching is based on the data provided by the patient regarding physical activity. Mobile apps are connected with validated devices through which the patient records his or her data (Smartwatch, Fitbit, etc.,). The system continuously monitors the patient's physical activity, keeping track of goals. The system provides warnings to the patient (e.g. low daily activity or excessive sedentariness in activities of daily living) (30).

Mobile apps also help patients in managing mental health problems and treatments supporting them to think differently. Apps make meditation easier by offering a series of audio lessons and programs that improve sleep, breathing exercises, relaxation, and mindful movement, to better cope with stress (31–34).

### Integrated care pathway

**Figure 4:**
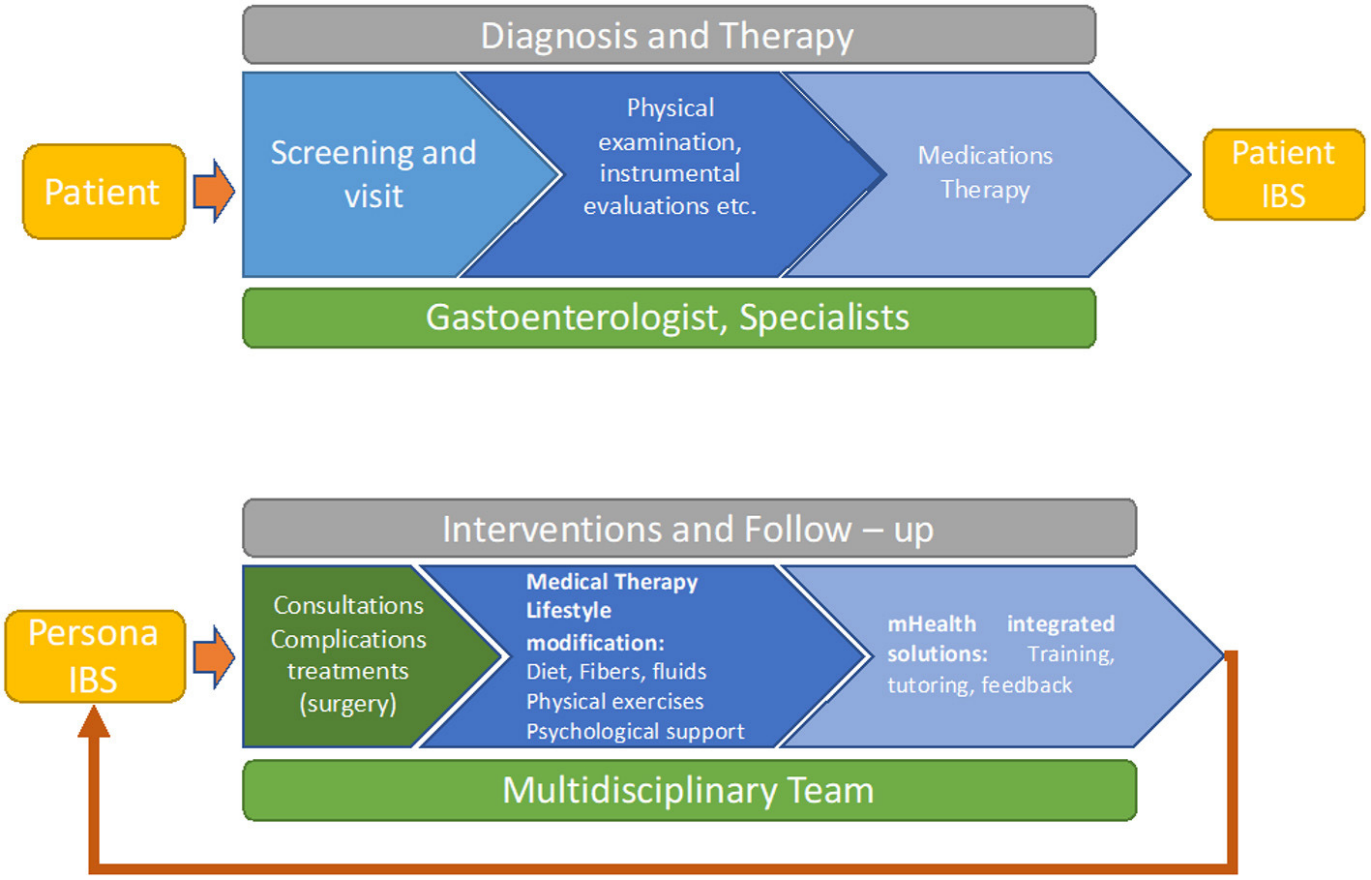
IBS integrated service process. **(Up)** Pathways to diagnosis. **(Down)** Pathways to health.

The process of mapping adapted the care pathway from the patient's perspective to improve quality of care and life and reduce costs. Once identified the gap in the care pathway it was possible to find specific options and or solutions to overcome them (Figure 4).

In the figure, we can see how two processes are activated for the patient. In the first, the patient must be diagnosed with a case of IBS and, once the necessary examinations have been performed, he or she must be given a treatment plan.

In the second, we see how the intervention and follow-up process starts with the definition of the Blueprint type of person in which the patient is to be placed. The multidisciplinary team, at this stage, will have to check whether surgical interventions are needed for possible complications. If not, the specialists in the team will be consulted, which will also happen after any surgical intervention. After the consultations, a treatment plan and a lifestyle change course will be prepared. Finally, integrated mHealth solutions will be implemented to support the patient and allow the team to have continuous information on the patients, preventing them from leaving the treatment pathway or having adverse episodes.

## Discussion

Patients affected by IBS usually include working age adults who complain about quality of life and the impact of disease reacutization on their work capacity. IBS symptoms are intertwined with stress, bio-psychological triggers and lifestyles, and mobile solutions provide the opportunity to facilitate service integration to address unmet IBS patient needs. Towards this goal, the “Personas” approach developed by the EU Blueprint on digital transformation of health and care combines quantitative methodologies and techniques with more synthetic and intuitive inference processes. This approach facilitates addressing the challenges and needs proposed by the design of digital experiences in an original way. Indeed, when developing services supported by digital solutions, it is important to consider the socio-economic context, skills and integration gaps, both technological and organizational, that may influence the adoption of innovative solutions: in our study we design an approach to personalize lifestyles support along the nutritional, physical and psychological domains, to integrate the diagnostic therapeutic pathway of IBS patients. Our approach is informed by the inputs emerging from a real outpatient practice but requires further validation through an implementation protocol.

To date, several dietary interventions have been proposed for the management of the IBS symptoms. Proper nutrition (4/5 meals a day at regular times), good hydration (1.5–2 L per day) and a reduced intake of substances such as insoluble fibres, alcohol, caffeine, spicy and fatty foods, as well as performing regular physical activity, are the main recommendations (35). However, ad alternative approach should be considered in cases where symptoms persist despite correct eating habits, and in this scenario, there is increasing evidence showing the beneficial effects of a low fermentable oligo-, di-, monosaccharides, and polyols (FODMAP) diet on IBS symptoms (36). In particular, FODMAP_S_ contained in some fruits, legumes, dairy products, and artificial sweeteners, can exacerbate symptoms due to their fermentation and osmotic effects in the lumen. These carbohydrates arrive at the level of the colon, where they induce the production of gas following the fermentation caused by the bacterial colic flora, with consequent luminal distension. Several observational studies have been conducted on the low-FODMAP diet, showing that this diet can significantly reduce abdominal pain, flatulence and diarrhoea in patients with IBS (37–39).

## Conclusions

The person-centered approach we implemented through the adaptation of the Blueprint persona methodology applied to IBS patients was implemented through a multidisciplinary Focus group that enabled the identification of the need for a mHealth intervention, based on the promotion of healthy lifestyles, nutrition, adapted physical activity, meditation practice and psychological support. Further research is needed to evaluate how mhealth services enable IBS patients to manage their conditions and change in the quality of life and be integrated into the current service provision flow. The integration of mHealth data with Electronic Health Record (EHR) and other data collected by professionals will allow identifying the minimum data set able to improve diagnosis and treatment. Feasibility, usability, and adaptability studies on innovative, integrated, personalized care paths, suitable for a wide range of patients, as well as cost studies, will highlight the impact of mHealth interventions on patients health outcomes, quality of life and possible reduction of health-related cost over time.

## Data availability statement

The raw data supporting the conclusions of this article will be made available by the authors, without undue reservation.

## Ethics statement

Ethical review and approval was not required for the study on human participants in accordance with the local legislation and institutional requirements. Written informed consent from the [patients/participants or patients/participants legal guardian/next of kin] was not required to participate in this study in accordance with the national legislation and the institutional requirements.

## Author contributions

MG: surgical observation and data collecting. VD: organization of the paper and drafting the manuscript. RP: processing data. DL: assessment of nutrional data. GT and MT: revision of the paper. LM: mapping the process. ES: assessment of psychiatric data. AC: project design. MI: project design and general organization. SL-R: critical revising. JF and RR-W: analysis of data. LK: revision of the manuscript and suggestions. Vigour consortium was approval and support. All authors contributed to the article and approved the submitted version.

## Conflict of interest

The authors declare that the research was conducted in the absence of any commercial or financial relationships that could be construed as a potential conflict of interest.

## Publisher's note

All claims expressed in this article are solely those of the authors and do not necessarily represent those of their affiliated organizations, or those of the publisher, the editors and the reviewers. Any product that may be evaluated in this article, or claim that may be made by its manufacturer, is not guaranteed or endorsed by the publisher.
